# Genetic Evaluation Weight, Carcass and Stayability in Nellore Females

**DOI:** 10.1111/jbg.12941

**Published:** 2025-05-08

**Authors:** Isabella Silva de Carvalho, Sirlene Fernandes Lázaro, Eula Regina Carrara, Matheus Rodrigues de Souza, Humberto Tonhati

**Affiliations:** ^1^ Faculdade de Ciências Agrárias e Veterinárias UNESP Jaboticabal Brazil; ^2^ Centre for Genetic Improvement of Livestock, Department of Animal Biosciences University of Guelph Guelph Ontario Canada; ^3^ The University of Georgia Athens Georgia USA

**Keywords:** animal breeding, genetic trends, GIBBS, heritability, selection efficiency

## Abstract

Traits related to growth, carcass quality and stayability are key components in enhancing the profitability and sustainability of Nelore cattle production systems. This study aimed to estimate heritabilities and genetic and environmental correlations for these traits using a Bayesian approach. Data from 94,703 females were analysed for weights at 210, 365 and 450 days of age (W210, W365 and W450), loin eye area (LEA), subcutaneous fat thickness in the loin (LFT) and rump (REFT) and stayability at 48, 54 and 72 months (STAY48, STAY54 and STAY72). Heritability estimates (± standard error) were 0.14 ± 0.03 for LEA, 0.20 ± 0.03 for LFT, 0.43 for REFT, 0.12 ± 0.02 for STAY54, and 0.18 ± 0.02 for STAY72. Moderate heritabilities for W210, W365, W450, LFT and REFT indicate a substantial additive genetic component, whereas lower estimates for LEA and stayability suggest a predominant influence of environmental factors. Genetic trends were generally positive but moderate: 0.14 kg/generation (W210), 1.40 kg/generation (W365), 1.77 kg/generation (W450), 0.016 cm^2^/generation (LEA) and 0.0081 months/generation (STAY72). In contrast, STAY48 showed a slightly negative trend (−0.0073 months/generation). Direct selection for W450 yielded a genetic gain of 9.837 kg, whereas indirect selection via correlated traits resulted in gains ranging from 0.125 to 9.272 kg. These findings highlight the relevance of environmental effects on traits with low heritability, such as LEA and stayability, and reinforce the effectiveness of selection for weight‐related traits due to their moderate heritability and favourable genetic trends.

## 1 Introduction

Weight at standardised ages and carcass traits (loin eye area (LEA), loin fat thickness (LFT) and rump fat thickness (REFT)) play a significant role in the selection of the Nelore breed. Also, stayability (STAY), that is, the animal's ability to survive up to a certain age in the herd, has a significant impact on the beef cattle production, directly affecting the economic return (Hamidi Hay and Roberts 2017) and influencing the profitability of other economically valuable traits, such as weight. It also helps reduce costs associated with the production of replacement heifers. STAY can be interpreted as an indicator of animal welfare, as high discard and mortality rates may indicate inadequate conditions of welfare, such as improper nutritional management, stressful and inappropriate handling, among others (Weigel et al. 2003). Selecting for STAY is crucial for applying genetic progress in other economically valuable traits, allowing for an increase in the number of animals available for selection and optimization of other traits (Oliveira et al. 2020). However, measuring STAY can be challenging, especially since it is often evaluated late in the animal's life or even after death. Thus, improving STAY, weight and carcass traits can increase profitability. The study aims: (1) to estimate components of (co)variance for adjusted weights for 210 (W210), 365 (W365) and 450 (W450) days of age, carcass traits (LEA, LFT and REFT) and stayability at 48 (STAY48), 54 (STAY54) and 72 (STAY72) months; (2) to evaluate the genetic trends of the traits; (3) to estimate genetic correlations among the traits; (4) to estimate genetic gains, correlated response and relative efficiency of selection for the traits.

## 2 Material and Methods

### 2.1 Phenotypic Data

The database refers to *Bos indicus* Nelore breed reproduction females, and it was composed of farm records, originating from the National Association of Breeders and Researchers (ANCP) database. The entire pedigree contained a total of 94,703 animals. Phenotypic quality control was performed to include only females with pedigree information. Traits were stayability at 48 months (STAY48), 54 months (STAY54) and the traditional at 72 months (STAY72). For STAY48 and STAY54, the females were challenged early. A value of 1 was assigned to failure, to display early females do not having three parturitions with corresponding weaned calves at the defined time period. A value of 2 was assigned to success to indicate the contrary, that is, females weaning at least three calves by the respective period. The STAY72 was related to success in giving birth to and weaning at least three calves (measured by the number of weaned progenies until 72 months); all females were included, not just the early ones, with one assigned to indicate failure (weaned fewer than three calves) and two indicating success (weaned three or more calves). Contemporary groups (CGs) were formed by combining the year of birth, the birth farm of the cow and the management group, previously defined by ANCP. Animals belonging to CGs with fewer than three records and records of animals without known parents were removed from the analysis. CGs, the linear and quadratic age of the dam (only for W210), and the age of the animal (only for LEA, LFT and REFT) were used as systematic effects. Table 1 presents the descriptive statistics of the data.

**TABLE 1:** Number of records (*N*) response categories classified as 1 (n1) and 2 (n2), coefficient of variation (CV [%]), maximum and minimum values obtained for each trait.

	*N*	Categories		CV (%)	Maximum	Minimum
		n1	n2			
STAY72	20,254	8843	11,411	31.72	2.00	1.00
STAY54	29,537	21,720	7817	34.88	2.00	1.00
STAY48	29,537	27,263	2274	24.75	2.00	1.00
W210 (kg)	18,506	—	—	11.33	295.00	99.00
W365 (kg)	20,572	—	—	12.81	479.00	159.00
W450 (kg)	21,733	—	—	12.78	526.00	188.00
LEA (cm^2^)	5968	—	—	6.81	69.00	54.00
LFT (mm)	7080	—	—	24.42	7.00	3.00
REFT (mm)	10,498	—	—	32.07	11.00	2.00

*Note:* For STAY48 and STAY54: n1 = failure (those that did not have three calvings with three weaned calves) and n2 = success (weaned at least three calves); for STAY72: n1 = failure (weaned less than three calves) and n2 = success (weaned three or more calves).

Abbreviations: LEA, rib eye area; LFT, subcutaneous fat thickness on the loin; REFT, subcutaneous fat thickness on the rump cap; STAY48, stayability at 48 months; STAY54, stayability at 54 months; STAY72, stayability at 72 months; W210, standardised weight at 210 days; W365, standardised weight at 365 days; W450, standardised weight at 450 days.

### 2.2 Statistical Analyses

Estimates of the variance components for single‐trait analyses were obtained with the BLUPF90 family of programmes (Misztal 2014), including phenotype and pedigree records. The models of analysis were:

(1) y=Χβ+Zu+Mmat+Pmpe+ε

(2) y=Χβ+Zu+ε

where *y* is the vector of phenotypes, *X* is the incidence matrix relating systematic effects to phenotypes, β is the vector of systematic effects, *Z* is the incidence matrix relating animals to phenotypes, *u* is the vector of direct additive genetic effects of each animal, *M* is the incidence matrix relating maternal additive effects to phenotypes, *P* is the incidence matrix relating permanent maternal environment effects to phenotypes, *mpe* is the vector of permanent maternal effects, *mat* is the vector of maternal additive effects, and 𝜀 is the vector of residuals. The distributions for u, *mpe*, ε and *y* were as follows:

$$u\sim N\left(0,G\right),\mathrm{mat}\sim N\left(0,M\right),\mathrm{mpe}\sim N\left(0,P\right),\varepsilon \sim N\left(0,R\right)$$

$$y\left|\beta \right.,u,\mathrm{mat},\mathrm{mpe},G,M,R∼N\left(\mathrm{Χ\beta}+\mathrm{Zu}+\text{Mmat}+\text{Pmpe},R\right)$$

where $G=A\sigma_{u}^{2}$, $M=A\sigma_{\mathrm{mat}}^{2}$, $P=I\sigma_{\mathrm{mpe}}^{2}$, $R=I\sigma_{\varepsilon }^{2}$, $\sigma_{u}^{2}$, $\sigma_{\mathrm{mat}}^{2}$, $\sigma_{\mathrm{mpe}}^{2}$ and $\sigma_{\varepsilon }^{2}$. Hence, the covariance matrices were for direct additive genetic, maternal, permanent maternal environment and residual variances, respectively. Matrix *A* is the numerator relationship matrix, and *I* is an identity matrix of order equal to the number of animals evaluated. Matrix G is the direct additive genetic variance matrix; P is the incidence matrix relating phenotype to permanent maternal environment; R is the residual variance matrix; *M* is the maternal additive genetic variance matrix.

For analysing STAY48, STAY54 and STAY72, a threshold model and categorical data analysis were employed (Mrode and Thompson 2005):

y=Χβ+Zu+ε

where y is the vector of thresholds on a normal scale, β and u are vectors of fixed and random effects, respectively. Χ is the incidence matrix relating phenotypes to systematic effects; *Z* is the incidence matrix relating animals to phenotypes, 𝜀 the vector of residuals. However, in the threshold model, it was assumed that the underlying scale has the following normal distribution:

$$u\;|\;\theta \sim N\;\left(\mathrm{W\theta},I\sigma_{e}^{2}\right)$$

where *u* is a vector of underlying scale with order *r* (where *r* is the number of animals); $\theta^{\prime }=\left(\beta^{\prime },a^{\prime }\right)$ is the parameter vector with order *s* (the number of effects in the model), where β is a vector of systematic effects (CG, linear and quadratic dam age (W210) and animal age (LEA, LFT and REFT)) with order *s*, and *a* is the additive genetic random effect; *W* is the incidence matrix with order *r* by *s*; *I* is the identity matrix with order equal to the number of animals evaluated, and $\sigma_{e}^{2}$ is the residual variance of the analysed traits.

The genetic parameters for all traits were estimated with a single‐trait threshold model using the Bayesian approach in the software GIBBSF90+ (Misztal 2014). For genetic correlations, multiple‐trait (i.e., three traits) analyses were carried out under a linear threshold model with an underlying Gaussian as follows.

1. Three‐trait model without including maternal effects (i.e., without W210):

(3) $$\left[\begin{array}{c} y_{1}\\ y_{2}\\ y_{3} \end{array}\right]=\left[\begin{array}{ccc} X_{1} & 0 & 0\\ 0 & X_{2} & 0\\ 0 & 0 & X_{3} \end{array}\right]\left[\begin{array}{c} \beta_{1}\\ \beta_{2}\\ \beta_{3} \end{array}\right]+\left[\begin{array}{ccc} Z_{1} & 0 & 0\\ 0 & Z_{2} & 0\\ 0 & 0 & Z_{3} \end{array}\right]\left[\begin{array}{c} u_{1}\\ u_{2}\\ u_{3} \end{array}\right]+\left[\begin{array}{c} e_{1}\\ e_{2}\\ e_{3} \end{array}\right]$$

2 Three‐trait model traits including maternal effects (i.e., with W210):

(4) $$\left[\begin{array}{c} y_{1}\\ y_{2}\\ y_{3} \end{array}\right]=\left[\begin{array}{ccc} X_{1} & 0 & 0\\ 0 & X_{2} & 0\\ 0 & 0 & X_{3} \end{array}\right]\left[\begin{array}{c} \beta_{1}\\ \beta_{2}\\ \beta_{3} \end{array}\right]+\left[\begin{array}{ccc} Z_{1} & 0 & 0\\ 0 & Z_{2} & 0\\ 0 & 0 & Z_{3} \end{array}\right]\left[\begin{array}{c} u_{1}\\ u_{2}\\ u_{3} \end{array}\right]+\left[\begin{array}{ccc} 0_{1} & 0 & 0\\ 0 & M_{2} & 0\\ 0 & 0 & 0_{3} \end{array}\right]\left[\begin{array}{c} 0_{1}\\ {\mathrm{mat}}_{2}\\ 0_{3} \end{array}\right]+\left[\begin{array}{ccc} 0_{1} & 0 & 0\\ 0 & C_{2} & 0\\ 0 & 0 & 0_{3} \end{array}\right]\left[\begin{array}{c} 0_{1}\\ {\mathrm{mpe}}_{2}\\ 0_{3} \end{array}\right]+\left[\begin{array}{c} e_{1}\\ e_{2}\\ e_{3} \end{array}\right]$$

In (1) and (2) $y_{1}$ was the vector of observations for W450; $y_{2}$ and $y_{3}$ were the vectors of observations for weight, carcass and threshold traits (W210, W365, LEA, LFT, REFT, STAY48, STAY54 and STAY72). The vector *β* contained the systematic non‐genetic effects (CG, dam age at calving as a linear and quadratic covariate (W210), and animal age (LEA, LFT and REFT)); *u* was the vector of direct additive genetic random effects; *mat* was the vector of maternal genetic effects (W210); 𝑚𝑝𝑒 is the vector of permanent maternal environmental effects (W210). The incidence matrices *X* and *Z* related the data with vectors *β* and *u*, respectively. The incidence matrix *M* related the phenotypes to maternal additive genetic effects (W210). Finally, *C* was the incidence matrix relating phenotypes to permanent maternal environmental effects for W210.

The Bayesian prior distributions were as follows: *β* = a uniform distribution (non‐informative prior); $u|\sum_{u}∼N\left(0,A⊗\sum_{u}\right)$, being *A* the numerator relationship matrix and $\sum_{u}$ is the direct additive genetic (co)variance matrix; $\mathrm{mat}|\sum_{\mathrm{mat}}∼N\left(0,A⊗\sum_{\mathrm{mat}}\right)$, $\sum_{\mathrm{mat}}$ was the maternal genetic (co)variance matrix; $\mathrm{mpe}|\sum_{\mathrm{mpe}}∼N\left(0,A⊗\sum_{\mathrm{mep}}\right)$, where *A* is the numerator relationship matrix of Wright's coefficients and $\sum_{\mathrm{mpe}}$ is the permanent maternal environment (co)variance matrix; $e|\sum_{e}∼N\left(0,I⊗\sum_{e}\right),$ the identity and residual (co)variance matrices, respectively. An inverse Wishart distribution was used as the prior distribution for all (co)variance matrices, $\sum_{u}$, $\sum_{\mathrm{mat}}$, $\sum_{\mathrm{mpe}}$ and $\sum_{e}$. The distributions for STAY48, STAY54 and STAY72 (with only two possible classes, 1 and 2) were assumed to have the following form

$$\begin{array}{c} f\left(\mathrm{yl},\beta ,u,\sum_{u},\sum_{\mathrm{mat}},\sum_{\mathrm{mpe}},\sum_{e},\tau \right)=\prod_{i=1}^{n}f\left(y_{i}\left|l_{i}\right.\right)\\ =\prod_{i=1}^{n}I\left(l_{i}<\tau \right)I\left(y_{i}=1\right)+\left(l_{i}>\tau \right)I\left(y_{i}=2\right) \end{array}$$

where τ is the threshold value that defines the two response categories (1 and 2).

Convergence of the Gibbs chains was performed by visual inspection and the criterion of Geweke (1992) by means of software “BOA” (Bayesian Output Analysis) written in R (Smith 2007). Geweke's (1992) method aims to compare the initial and final values of the Markov chain to detect potential issues with the convergence. In this regard, the null hypothesis states that convergence occurs non‐significantly (*p* > 0.05) between tested values to support this assertion. Probability values smaller than 0.05 suggest that the chains did not converge.

The number of iterations of the Markov Chain Monte Carlo (MCMC) analysis for single trait models was 500,000 (i.e., for LEA, LFT and REFT), and 750,000 for W210, W365, W450, STAY48, STAY54 and STAY72. We took a burn‐in period ranging from 100,000 (LEA, LFT and REFT) to 200,000 (W210, W365, W450, STAY48, STAY54 and STAY72) and a thinning interval of 100 cycles. For all three‐trait analyses, 1,000,000 to 1,500,000 iterations of the Markov Chain Monte Carlo (MCMC) simulation were used, with a burn‐in period of 500,000 to 750,000 and a thinning interval of 100 to 250 cycles.

### 2.3 Genetic Trend

To evaluate the genetic trend of the traits studied over the years of selection, the estimated breeding values (EBV) were calculated using the BLUPF90+ software (Misztal 2014). Genetic trends were estimated by regressing the animal's genetic value on its generation (Brinks et al. 1961) under the following model:

$$Y_{i}=b_{1}x_{1}+b_{0}+\varepsilon_{i}$$

where: $Y_{i}$ is the breeding value of the evaluated traits for the *i*th generation; $b_{1}$ is the slope coefficient; $x_{1}$ is the *i*th generation; $b_{0}$ is the intercept; $\varepsilon_{i}$ is the random error.

### 2.4 Genetic Gain and Correlated Response

Genetic gain and expected correlated response were calculated for STAY traits and W450 with weight and carcass traits, considering the same selection intensity (1.0). The formulas used can be represented as follows:

$$\begin{array}{c} {\mathrm{\Delta G}}_{k}=i.\sigma_{\mathrm{pk}}.h^{2}k,{\mathrm{RC}}_{k-j}\\ =r_{\mathrm{ak}-j}.h_{k}.h_{j}.i.\sigma_{\mathrm{pj}},{\mathrm{ER}}_{k-j}=\left(\frac{{\mathrm{RC}}_{k-j}}{{\mathrm{\Delta G}}_{k}}\right)x100 \end{array}$$

where ${\mathrm{\Delta G}}_{k}$ was the predicted genetic gain based on direct selection for the traits of interest, where *k* refers to the traits under selection (W450, STAY48, STAY54 and STAY72); i is the selection intensity; $\sigma_{\mathrm{pk}}$ is the phenotypic standard deviation of the trait under selection; $h2_{k}$ is the heritability of the trait under selection; *j* refers to the traits LEA, LFT, REFT (carcass), W210, W365 (weight) and STAY48, STAY54 and STAY72 (stayability); ${\mathrm{RC}}_{k-j}$ is the correlated response obtained for W450 and the different STAYs through indirect selection for LEA, LFT, REFT, W210, W365, STAY48, STAY54 and STAY72; $r_{\mathrm{ak}-j}$ is the genetic correlation between the traits under selection and the weight (W210 and W365), carcass (LEA, LFT and REFT) and the different STAYs; $h_{j}$ and $h_{k}$ are the square roots of the heritability $\left(h^{2}\right)$ of *j* and *k*, respectively; $\sigma_{p_{j}}$ is the phenotypic standard deviation of *j*; ${\mathrm{ER}}_{k-j}$ refers to the relative efficiency of indirect selection for W450 and the different STAYs compared to the direct response for W450 and the different STAYs.

## 3 Results and Discussion

Variance components, heritabilities, posterior standard deviations and 95% posterior probability density intervals (HPD95) are shown in Table 2. It is important to note that the Geweke test did not reveal significance (*p* > 0.05), not rejecting the convergence of the MCMC algorithm.

**TABLE 2:** Posterior means, posterior standard deviations (PSD), and 95% highest posterior density intervals (HPD95) for variance components, animal heritability (h^2^a) and maternal heritability (h^2^m) were obtained for the traits evaluated in Nelore cattle.

	$\sigma_{\mathrm{gd}}^{2}$	$\sigma_{m}^{2}$	$\sigma_{e}^{2}$	$\sigma_{p}^{2}$	$h_{a}^{2}$	$h_{m}^{2}$
W210	91.870 (12.110)	34.15 (6.04)	167.500 (8.400)	305.350 (6.050)	0.300 (0.040)	0.110 (0.020)
	[67.390; 114.300]	[22.72;46.08]	[150.600; 183.700]	[293.290; 316.670]	[0.220; 0.370]	[0.070; 0.150]
W365	198.220 (15.850)	—	333.180 (11.530)	531.390 (7.060)	0.370 (0.030)	—
	[167.500; 229.500]	—	[311.100; 356.800]	[517.900; 545.400]	[0.320; 0.420]	—
W450	243.530 (17.060)	—	360.740 (12.160)	604.270 (8.020)	0.400 (0.020)	—
	[211.100; 277.500]	—	[336.000; 383.400]	[588.600; 619.800]	[0.330; 0.430]	—
LEA	1.800 (0.450)	—	11.390 (0.410)	13.190 (0.280)	0.140 (0.030)	—
	[1.010; 2.750]	—	[10.550; 12.170]	[12.650;13.730]	[0.070;0.200]	—
LFT	0.200 (0.030)	—	0.790 (0.030)	0.990 (0.020)	0.200 (0.030)	—
	[0.130; 0.270]	—	[0.730; 0.840]	[0.960; 1.030]	[0.140; 0.260]	—
REFT	1.080 (0.090)	—	1.420 (0.070)	2.500 (0.050)	0.430 (0.030)	—
	[0.880; 1.250]	—	[1.280; 1.550]	[2.410;2.590]	[0.360; 0.490]	—
STAY48	0.130 (0.030)	—	1.000 (0.010)	1.130 (0.030)	0.110 (0.020)	—
	[0.070; 0.190]	—	[0.980;1.020]	[1.070; 1.200]	[0.070;0.160]	—
STAY54	0.140 (0.020)	—	1.010 (0.020)	1.150 (0.030)	0.120 (0.020)	—
	[0.100; 0.190]	—	[0.990; 1.030]	[1.100;1.210]	[0.090; 0.160]	—
STAY72	0.018 (0.002)	—	0.08 (0.003)	0.100 (0.003)	0.180 (0.020)	—
	[0.010; 0.020]	—	[0.080; 0.090]	[0.100; 0.120]	[0.130; 0.230]	—

Abbreviations: $h_{a}^{2}$, animal additive genetic heritability; $h_{m}^{2}$, maternal genetic heritability; $\sigma_{e}^{2}$, residual variance; $\sigma_{\mathrm{gd}}^{2}$, direct genetic variance; $\sigma_{m}^{2}$, maternal genetic variance; $\sigma_{p}^{2}$, phenotypic variance; LEA, loin eye area; LFT, subcutaneous fat thickness in the loin; REFT, subcutaneous fat thickness in the rump cap; STAY48, stayability at 48 months; STAY54, stayability at 54 months; STAY72, stayability at 72 months; W210, standardised weight at 210 days; W365, standardised weight at 365 days; W450, standardised weight at 450 days.

### 3.1 Heritability Estimates

Heritability estimates obtained through the uni‐trait model for W210, W365, W450, LFT, REFT, LEA, STAY48, STAY54 and STAY72 suggest that there is additive genetic variability in these traits. A narrower range for HPD95 was observed for W365, W450, STAY48, STAY54 and STAY72, suggesting relative certainty regarding the parameter estimate, while for W210, LEA, LFT and REFT, HPD95 had a wider range, indicating greater uncertainty. The estimated heritability for W210 was 0.30. Similar results were reported in the literature, showing variation from 0.11 to 0.39 in Nelore cattle (Zuin et al. 2012; Lira et al. 2013; Oliveira et al. 2017; Kluska et al. 2018; Santos et al. 2019; Bessa et al. 2021). The estimated maternal heritability for W210 was 0.11, consistent with that reported by Oliveira et al. (2017) (0.12) using random regression models in Nelore cattle, suggesting that despite the low magnitude, maternal breeding value is manifested in the weaning weight of the offspring of this female, contributing to the phenotypic variation of W210.

For W365, the estimated heritability was 0.37. Lira et al. (2013) reported an estimated heritability of 0.41 in Nelore using a single‐trait model, while Evangelista et al. (2020) obtained an estimate of 0.46 in polled Nelore from a multiple‐trait model. The results obtained in the current study were lower than those reported by those authors, which could be explained by the larger amount of information in both studies. Conversely, Santos et al. (2019), using a uni‐trait model in Nelore, obtained heritability estimates ranging from 0.11 to 0.17 for W365.

The estimated heritability for W450 was 0.40, suggesting that there is also sufficient genetic variability to allow genetic progress for this trait, although with moderate gains per generation. A higher estimate (0.44) was reported by Kluska et al. (2018) in Nelore using genomic information.

For REFT, the estimated heritability was 0.43, suggesting that a significant portion of the genetic variation in these traits is influenced by additive genes, which directly contribute to the animal's phenotype in an additive manner. Lower values ranging from 0.19 to 0.32 have been reported by Lima Neto et al. (2009), Barbosa et al. (2010), Faria et al. (2015), Paula Junior et al. (2015) and Buzanskas et al. (2017). However, Barbosa et al. (2010) reported a higher estimated heritability (0.65) in Nelore; this difference could be explained by the use of males by these authors, while the present study used only female information.

The estimated posterior distribution of heritability for LEA was 0.14, which would suggest a lower response to selection for this trait. However, given the economic importance of this trait, its use in genetic improvement programmes is still justified. Gordo et al. (2016) found a slightly lower estimate (0.13). Higher values were also found in the literature for Nelore ranging from 0.25 to 0.41 (Bonin et al. 2015; Paula Junior et al. 2015; Buzanskas et al. 2017). Lima Neto et al. (2009) estimated a value of 0.35 from single‐trait analysis in the Guzerá breed, whose herd consisted of 90% males and about 10% females, indicating that when LEA is measured in males, heritability may be higher because the improvement in LEA will be better visualised in the bull descendants of these females, since males are preferred for slaughter and females used as breeding dams.

STAY is measured late in the life of the beef animal, and it is limited by sex, which may result in low genetic progress. The heritability estimate for STAY48 was 0.11. Queiroz et al. (2007) reported an estimate of 0.28 for the ability to stay at 48 months using a Bayesian method from Caracu animals. Silva et al. (2021) found a 0.10 estimate for STAY48 employing multiple‐trait models. The difference in values found between these studies is based on the different environmental conditions and management practices among populations. For STAY54, heritability was equal to 0.12. Specific studies with STAY54 could not be found because of the use of STAY52 (Ramos et al. 2021), STAY53 (Ramos et al. 2019), STAY60 (Silva et al. 2016; Nascimento et al. 2023), STAY63 (Ramos et al. 2019), STAY64 (Ramos et al. 2019). Ramos et al. (2021) working with STAY52 in Nelore cattle using the Bayesian method, reported an estimate of 0.092, similar to the present study.

The heritability estimate for STAY72 was 0.18. Nascimento et al. 2023 reported similar estimates in Jersey cattle (0.15 to 0.18), and a slightly higher estimate (0.23) was reported in Caracu animals (Queiroz et al. 2007). The heritability for STAY72 (0.19) estimated in this study falls within the values reported by other authors using STAY76 in Nelore and Brahman animals, 0.14 to 0.19 (Kluska et al. 2018; Schmidt et al. 2018; Ramos et al. 2019, 2021; Bessa et al. 2021). The low heritability estimates attributed to STAY48, STAY54 and STAY72 suggest that variability in these traits may be predominantly influenced by environmental factors such as climate, which directly affects the availability of forage, management, nutrition, welfare and/or non‐additive genetic effects.

### 3.2 Genetic Trends

**FIGURE 1:**
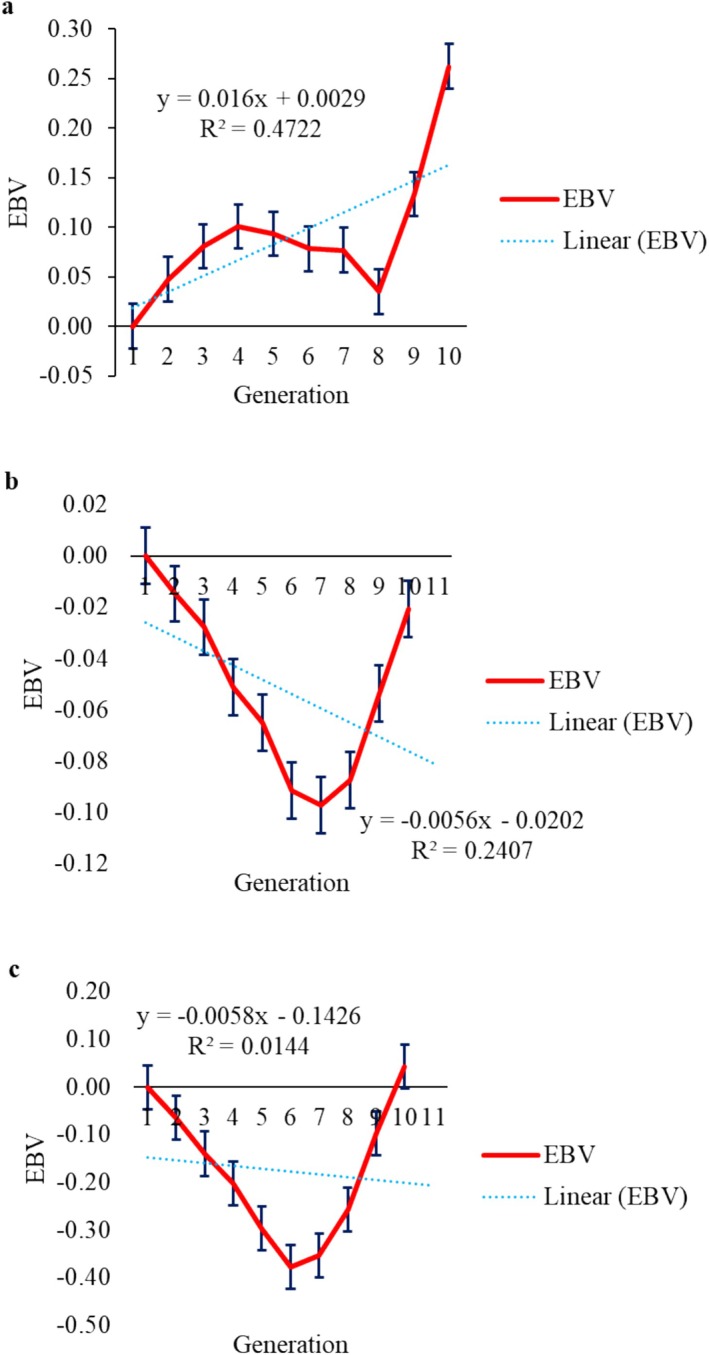
Genetic trend (linear regression) for ribeye area (LEA, a), backfat thickness (LFT, b) and rump fat thickness (REFT, c). [Colour figure can be viewed at wileyonlinelibrary.com]

**FIGURE 2:**
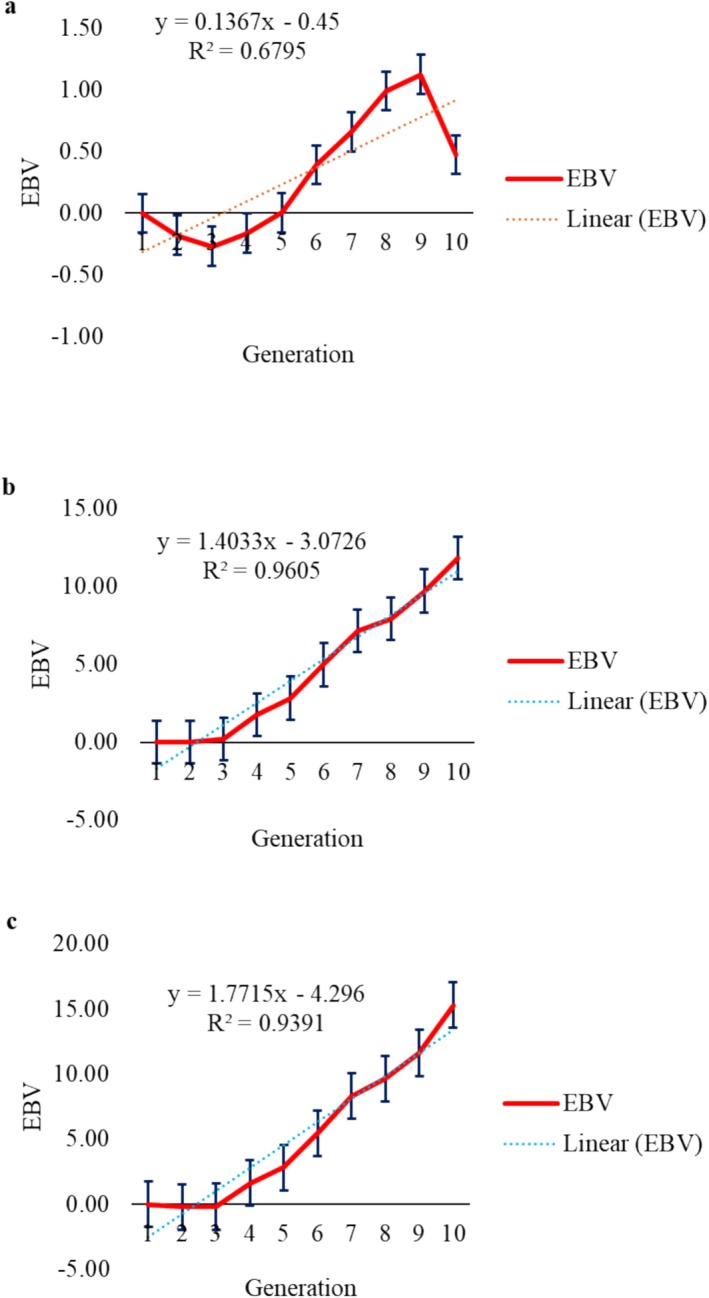
Genetic trend (linear regression) for weight at 210 days (W210, a), weight at 365 days (W365, b) and weight at 450 days (W450, c). [Colour figure can be viewed at wileyonlinelibrary.com]

**FIGURE 3:**
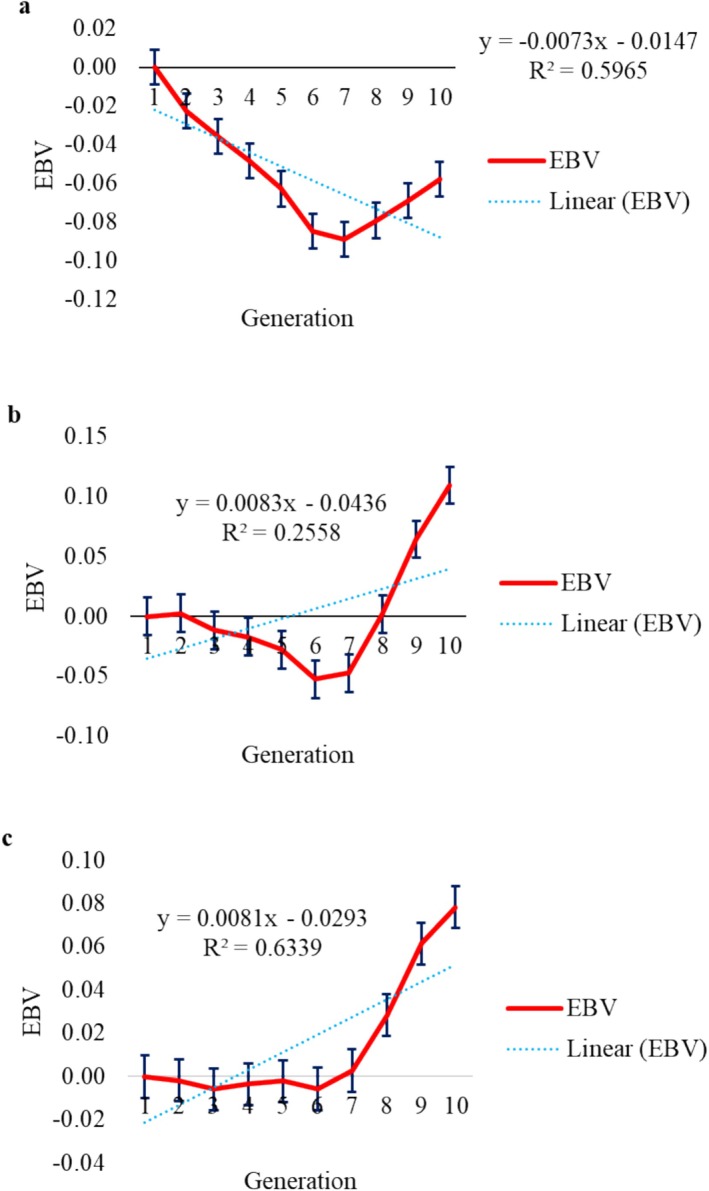
Genetic trend (linear regression) for stayability at 48 months (STAY48, a), stayability at 54 months (STAY54, b), stayability at 72 months (STAY72, c). [Colour figure can be viewed at wileyonlinelibrary.com]

Genetic trend allows observing changes in traits over time when driven by the selection process. Genetic trends were estimated by regressing genetic values on the generation coefficients of the animals and were significant (*p* < 0.05) for LEA (Figure 1a), W210 (Figure 2a), W365 (Figure 2b), W450 (Figure 2c), STAY48 (Figure 3a) and STAY72 (Figure 3c), equal to 0.016 cm^2^/generation, 0.14 kg/generation, 1.40 kg/generation, 1.77 kg/generation, −0.0073 months/generation and 0.0081 months/generation, respectively. For LFT (Figure 1b), REFT (Figure 1c) and STAY54 (Figure 3b), genetic trends were not significant (*p* > 0.05) and equal to −0.006 mm/generation, 0.006 mm/generation and 0.0083 months/generation, respectively.

For LEA, the genetic trend was 0.016 cm^2^/generation. Studies using regression of genetic values on generation coefficients are scarce; therefore, the studies mentioned here regressed genetic values on the birth year of the animals. Menezes et al. (2013) reported a higher genetic trend estimate of 0.012 cm^2^/year, while Bessa et al. (2021) reported a lower estimate of 0.012 cm^2^/year. This is a trait of great importance for the beef packing industry, where males predominate and deserves attention. In this study, the analysis focused on Nelore females, explaining the decreasing trend over the years studied, while possible improvements will become clearer in their progeny. The genetic trend relative to LEA is low, but it could be improved by incorporating data from the entire herd and not just from females.

For LFT, the genetic trend was low and negative (−0.006 mm/generation), indicating an unfavourable trend for this trait, while for REFT, the genetic trend was positive (0.006 mm/generation), indicating a favourable trend. Both traits are directly related to meat quality, as they help prevent toughening caused by dehydration and cooling during the industrial process. Similar but positive values using regression on birth year for LFT have been reported in the literature for both Nelore and Brahman cattle, ranging from 0.003 to 0.0304 mm/year (Silva et al. 2009; Menezes et al. 2013; Bessa et al. 2021). Bessa et al. (2021) estimated a value of 0.010 mm/year for REFT in Brahman cattle, which is higher than the value found in the present study. The coefficient of determination (*R* ^2^) was higher for LEA (0.47), indicating that the proposed model for this trait explained the variation in the data better compared to LFT (0.24) and REFT (0.01), whose *R* ^2^ values were lower, and in the case of REFT, close to zero.

For W210, the trend (0.07 kg/generation) is positive and consistent with values found in the literature ranging from 0.016 to 0.1870 kg/year (Amaral et al. 2014; Oliveira et al. 2017). The standardised weight between 205 and 240 days stands out as a merit for cattle breeding systems, serving as a selection criterion and can be employed at the moment of culling and selection of animals, allowing the choice of those that best align with the system and production objectives. For W365, the genetic trend was 1.40 kg/generation, indicating a significant increase in breeding values over the generations. This trait shows an upward trajectory in genetic trend. Zuin et al. (2012) reported a lower trend in Nelore cattle of 0.078 kg/year. Souza et al. (2011) estimated genetic trends for Nelore cattle of 0.80 kg/year, and Bessa et al. (2021) reported a value of 0.59 kg/year in Brahman cattle.

The genetic trend for W450 was 1.77 kg/generation, outlining a clear trend of growth in genetic values over the years. Bessa et al. (2021) reported a value of 0.70 kg/year in Brahman cattle. Menezes et al. (2022) found a value of 0.13 kg/year in Caracu cattle at 500 days. The *R* ^2^ was greater than 0.90 for W365 and W450, indicating that the proposed model for the ponderal traits explains the variation in the data.

Regarding STAY48, the genetic trend estimate was −0.0073 months/generation, pointing to limitations in its ability to explain the variability of the birth year related to STAY48. For STAY54 and STAY72, genetic trends were positive (0.0043 mm/generation and 0.0083 months/generation, respectively), suggesting that the year exerts a statistically significant influence on this trait. For STAY72, Bessa et al. (2021) estimated a trend of −0.05 days/year in Brahman cattle. The analysis indicates that factors beyond genetics may have a substantial impact, emphasising the need for a more comprehensive analysis which accounts for other environmental variables, especially those related to management. The value of *R* ^2^ was higher for STAY72 (0.63), suggesting that the proposed model accounted for more variability when compared to STAY48 (0.59) and STAY54 (0.26).

### 3.3 Genetic Correlation Estimates

The genetic correlations, posterior standard deviations, and 95% posterior probability density intervals (HPD95) are presented in Table 3.

**TABLE 3:** Genetic correlation estimates and residual correlations with their respective standard deviations (SD) and highest posterior density intervals (HPD95, in brackets) obtained through multi‐trait analyses.

Multi‐trait model								
Traits			$r_{g_{12}}$	$r_{g_{13}}$	$r_{g_{23}}$	$r_{e_{12}}$	$r_{e_{13}}$	$r_{e_{23}}$
1	2	3						
W450	STAY48	W365	−0.278 (0.103)	0.986 (0.005)	−0.324 (0.100)	0.173 (0.028)	0.814 (0.006)	0.150 (0.027)
			[−0.474; −0.090]	[0.978;0.995]	[−0.506; −0.129]	[0.120; 0.229]	[0.801; 0.827]	[0.096; 0.200]
		LEA	−0.279 (0.108)	0.182 (0.117)	0.036 (0.152)	0.165 (0.028)	0.308 (0.034)	−0.015 (0.032)
			[−0.481; 0.055]	[−0.047;0.413]	[−0.240; 0.363]	[0.111; 0.219]	[0.244; 0.374]	[−0.076; 0.049]
		LFT	−0.302 (0.118)	0.019 (0.101)	0.259 (0.131)	0.166 (0.034)	0.164 (0.033)	0.035 (0.031)
			[−0.524; −0.065]	[−0.170;0.211]	[0.018; 0.514]	[0.106; 0.221]	[0.101; 0.230]	[−0.023; 0.097]
		REFT	−0.270 (0.110)	0.023 (0.063)	0.272 (0.108)	0.158 (0.028)	0.242 (0.032)	0.071 (0.032)
			[−0.488; −0.072]	[−0.102;0.145]	[0.072; 0.482]	[0.106; 0.215]	[0.181; 0.307]	[0.007; 0.132]
		STAY54	−0.330 (0.102)	−0.114 (0.085)	0.838 (0.054)	0.158 (0.028)	0.085 (0.023)	0.987 (0.005)
			[−0.512; −0.119]	[−0.285;0.048]	[0.737;0.942]	[0.103; 0.210]	[0.041; 0.131]	[0.976; 0.997]
		STAY72	−0.283 (0.156)	0.029 (0.097)	0.599 (0.323)	0.168 (0.076)	0.074 (0.082)	0.980 (0.041)
			[−0.554;‐0.001]	[−0.142;0.224]	[−0.405; 0.848]	[0.091; 0.264]	[−0.020;0.162]	[0.969; 0.999]
W450	STAY54	W365	−0.176 (0.079)	0.986 (0.005)	−0.221 (0.079)	0.101 (0.022)	0.814 (0.006)	0.107 (0.021)
			[−0.319; −0.018]	[0.977;0.995]	[−0.372; −0.070]	[0.058; 0.142]	[0.801; 0.826]	[0.064;0.145]
		LEA	−0.129 (0.087)	0.183 (0.118)	−0.001 (0.129)	0.088 (0.022)	0.309 (0.034)	0.020 (0.025)
			[−0.294;0.043]	[−0.041;0.425]	[−0.246; 0.254]	[0.045; 0.131]	[0.244; 0.376]	[−0.027; 0.073]
		LFT	−0.147 (0.089)	0.027 (0.101)	0.149 (0.108)	0.092 (0.023)	0.162 (0.033)	0.037 (0.024)
			[−0.317; 0.029]	[−0.162;0.220]	[−0.074; 0.356]	[0.045; 0.135]	[0.097; 0.227]	[−0.011;0.084]
		REFT	−0.131 (0.087)	0.030 (0.063)	0.168 (0.088)	0.088 (0.022)	0.239 (0.032)	0.080 (0.026)
			[−0.300; 0.036]	[−0.098;0.150]	[−0.011; 0.327]	[0.044; 0.130]	[0.176; 0.304]	[0.030; 0.131]
		STAY72	−0.157 (0.098)	0.079 (0.097)	0.823 (0.130)	0.087 (0.026)	0.069 (0.033)	0.997 (0.002)
			[−0.351; 0.032]	[−0.113;0.259]	[0.465; 0.938]	[0.039; 0.141]	[0.006; 0.131]	[0.993; 1.000]
W450	STAY72	W365	0.009 (0.079)	0.985 (0.004)	0.011 (0.080)	0.090 (0.027)	0.815 (0.006)	0.081 (0.026)
			[−0.139; 0.171]	[0.978;0.993]	[−0.152; 0.163]	[0.037; 0.144]	[0.802; 0.827]	[0.029; 0.131]
		LEA	0.032 (0.085)	0.171 (0.105)	−0.034 (0.134)	0.092 (0.028)	0.311 (0.032)	0.062 (0.034)
			[−0.128; 0.198]	[−0.044;0.367]	[−0.283; 0.243]	[0.039; 0.146]	[0.251; 0.377]	[−0.003; 0.130]
		LFT	0.026 (0.085)	0.019 (0.098)	0.042 (0.127)	0.094 (0.028)	0.165 (0.032)	0.061 (0.034)
			[−0.137; 0.194]	[−0.171;0.206]	[−0.224; 0.276]	[0.042; 0.151]	[0.100;0.227]	[−0.005;0.129]
		REFT	0.011 (0.089)	0.025 (0.062)	0.108 (0.089)	0.098 (0.029)	0.241 (0.032)	0.083 (0.034)
			[−0.173;0.176]	[−0.097;0.147]	[−0.063; 0.278]	[0.042; 0.157]	[0.180; 0.304]	[0.016; 0.147]
W450	W365	LEA	0.985 (0.004)	0.228 (0.107)	0.221 (0.108)	0.814 (0.007)	0.299 (0.033)	0.271 (0.031)
			[0.977; 0.992]	[0.013;0.425]	[0.003; 0.416]	[0.801; 0.827]	[0.234; 0.362]	[0.211; 0.334]
		LFT	0.985 (0.005)	0.009 (0.094)	0.011 (0.093)	0.815 (0.007)	0.177 (0.032)	0.156 (0.031)
			[0.978; 0.996]	[−0.174; 0.188]	[−0.167; 0.189]	[0.802; 0.828]	[0.115; 0.240]	[0.096; 0.215]
		REFT	0.984 (0.005)	0.018 (0.062)	0.037 (0.064)	0.817 (0.006)	0.252 (0.032)	0.213 (0.031)
			[0.975; 0.993]	[−0.106; 0.131]	[−0.095; 0.156]	[0.803; 0.829]	[0.185; 0.312]	[0.152; 0.277]
W450	LEA	LFT	0.170 (0.110)	−0.005 (0.095)	−0.193 (0.134)	0.307 (0.032)	0.160 (0.032)	0.154 (0.031)
			[−0.041; 0.386]	[−0.193; 0.179]	[−0.455; 0.072]	[0.242; 0.368]	[0.094; 0.222]	[0.097; 0017]
		REFT	0.193 (0.113)	0.025 (0.061)	−0.040 (0.104)	0.304 (0.032)	0.230 (0.031)	0.168 (0.033)
			[−0.018; 0.410]	[−0.094; 0.142]	[−0.237; 0.148]	[0.241; 0.368]	[0.170; 0.292]	[0.106; 0.235]
W450	LFT	REFT	0.082 (0.081)	0.033 (0.062)	0.660 (0.058)	0.173 (0.032)	0.235 (0.031)	0.405 (0.027)
			[−0.084; 0.237]	[−0.089; 0.162]	[0.545; 0.773]	[0.112; 0.237]	[0.174; 0.298]	[0.351; 0.456]

Abbreviations: $r_{e_{12}}$, residual correlation between W450 and W210, W365, LEA, LFT and REFT; $r_{e_{13}}$, residual correlation between W450 and traits 3 (W210, W365, STAY48, STAY54, STAY72, LEA, LFT and REFT); $r_{e_{23}}$, residual correlation between traits 2 (STAY48, STAY54, STAY72, W365, LEA and LFT) and traits 3 (W210, W365, STAY48, STAY54, STAY72, LEA, LFT and REFT); $r_{g_{12}}$, genetic correlation between W450 and W210, W365, LEA, LFT and REFT; $r_{g_{13}}$, genetic correlation between W450 and traits 3 (W210, W365, STAY48, STAY54, STAY72, LEA, LFT and REFT); $r_{g_{23}}$, genetic correlation between traits 2 (STAY48, STAY54, STAY72, W365, LEA and LFT) and traits 3 (W210, W365, STAY48, STAY54, STAY72, LEA, LFT and REFT); LEA, loin eye area; LFT, subcutaneous fat thickness in the loin; REFT, subcutaneous fat thickness in the rump; STAY48, stayability at 48 months; STAY54, stayability at 54 months; STAY72, stayability at 72 months; W210, standardised weight at 210 days; W365, standardised weight at 365 days; W450, standardised weight at 450 days.

The estimated genetic correlations between STAY48‐STAY54, STAY48‐STAY72, STAY54‐STAY72, W450‐W365 and LFT‐REFT were high. The genetic correlations between W450‐STAY48, W450‐STAY54, STAY48‐W365, STAY72‐LEA and LEA‐LFT were negative and of low magnitude (−0.330 to −0.193). Conversely, the genetic correlations between W450‐LEA were positive and of low magnitude (0.171 to 0.228). Kluska et al. (2018) reported lower results than those found in the present study (0.04).

Overall, W450 was more strongly correlated with W365, LEA, LFT, STAY48, STAY54 and STAY72 (−0.330 to 0.986) compared to REFT (0.018 to 0.033). The genetic correlations between STAY48, STAY54, STAY72 and LFT with W450 revealed negative values, while the genetic correlation estimates of W450 with LEA were positive and of low magnitude, indicating that selection to increase LEA may result in a slight increase in W450. In the context of W450, the lowest genetic correlation reported was W450‐REFT (0.018), indicating virtually null association. On the other hand, W365 correlated more strongly with LEA (0.228) when compared with LFT and REFT (0.009 and 0.018, respectively).

Pleiotropy, where a single gene affects multiple phenotypic traits, can justify these results, as the genetic correlation matrix reflects these interrelationships (Kirkpatrick 2009; Zhang 2023). In this regard, higher eigenvalues indicate significant genetic variation in a given direction, thus suggesting stronger pleiotropy. On the other hand, low eigenvalues indicate limited genetic variation. Genetic correlations between traits under selection often constrain selection response, particularly in multiple trait scenarios (Kirkpatrick 2009). As the number of traits increases, genetic interactions become more complex, creating constraints that reduce selection efficiency. However, the average response tends to decline gradually due to residual genetic variation and the nature of the interactions, allowing some progress (Kirkpatrick 2009). These findings support the theory that genetic correlations influence selection efficiency, impacting genetic progress and population adaptability in programmes involving multiple traits.

Overall, STAY48 was more strongly correlated with W365, W450, LFT, REFT, STAY54 and STAY72 (−0.324 to 0.838) compared to LEA (0.036). The genetic correlations between W365 and W450 with STAY48 revealed negative values, while the genetic correlation estimates of STAY48 with LFT, REFT and STAY72 were positive and of low magnitude, indicating that selection to increase LFT, REFT and STAY72 may result in a slight increase in STAY48. Conversely, STAY54 correlated more strongly with W365, W450, REFT, STAY54 and STAY72 (−0.372 to 0.823) compared to LEA (−0.001). For STAY72, stronger correlations were estimated with REFT (0.108). The genetic correlation estimates between STAY72‐W365 and STAY72‐LEA revealed negative values, and the correlation between STAY72 and LFT was positive and low, indicating that selection to increase LFT may result in a slight increase in STAY72.

For LEA, the strongest correlation was estimated with LFT (−0.193), revealing a negative value, indicating that selection to increase LFT may result in a slight reduction in STAY72. The genetic correlation between LFT and REFT was positive and of high magnitude (0.660), as they are traits that are related to each other; thus, selecting for LFT will lead to an increase in REFT.

Other genetic correlations of low magnitude were found between W450 and STAY48 and STAY54 (−0.330 to −0.129), STAY48 and W365 (−0.324), STAY54 and W365 (−0.221), showing a similar pattern among them. The results suggest that selection for increasing ponderal (Kluska et al. 2018) traits would have a limited impact on STAYs. Buzanskas et al. (2010) and Santana Júnior et al. (2013) reported correlations close to zero between STAY72 and yearling weight (W365, W450, P550), ranging from −0.02 to −0.09 for Nelore and Canchim cattle, respectively, while positive correlations were reported, ranging from 0.01 to 0.18 (Silva II et al. 2003; Kluska et al. 2018; Ramos et al. 2021).

Regarding STAY48 associated with LEA, a positive genetic correlation of low magnitude marginally close to zero (0.036) was observed. Kluska et al. (2018) reported a genetic correlation of 0.05. In contrast, the associations between LEA and STAY54 and STAY72 revealed negative values of low magnitude, ranging posteriori from (−0.001 to −0.034, respectively), being practically null, results close to the value found by Boldt et al. (2017) of 0.01.

The residual correlation estimates obtained for W450 associated with W365, LEA, LFT, REFT, STAY48, STAY54 and STAY72 ranged from 0.074 to 0.817. The residual correlations for the STAYs associated with each other and with LEA, LFT, REFT and W365 ranged from −0.015 to 0.997. These results were also similar to those reported previously for Nelore cattle (Zuin et al. 2012; Caetano et al. 2013; Almeida 2022). For W365 associated with LEA, LFT and REFT, they were positive and of low magnitude (0.156 to 0.271), and for LEA‐LFT, LEA‐REFT and LFT‐REFT, they ranged from (0.154 to 0.405). This indicates that the effect of residual variance should be considered in the model. Overall, genetic correlations were stronger than residual correlations. The low to high magnitude of genetic and residual correlations indicates that similar factors, such as management and nutrition, affect production similarly (Lázaro et al. 2021).

Genetic and residual correlations for W450 associated with W210 were not obtained because the data did not converge during the analysis, suggesting that the model was not sufficient to explain the relationship between W210 and the other traits under analysis.

### 3.4 Genetic Gains and Correlated Response

Genetic gains for W450, through indirect selection for W365, LEA, LFT, REFT, STAY48, STAY54 and STAY72; STAY48, through indirect selection for W210, W365, LEA, LFT, REFT, STAY54 and STAY72; STAY54 through indirect selection for W210, W365, LEA, LFT, REFT and STAY72; STAY72 through indirect selection for W210, P35, LEA, LFT and REFT, as well as relative selection efficiency, are presented in Table 4.

**TABLE 4:** Genetic gain estimates in months (ΔG), correlated response (RC) and relative selection efficiency (ER%) for W450, STAY (48, 54 and 72) in relation to LEA, LFT, REFT, W210, W365 and STAY48, STAY54 and STAY72.

	TRAITS	ΔG (W450)	RC	ER (%)
W450	LFT	9.837	0.327	3.323
	REFT	9.837	0.262	2.664
	LEA	9.837	1.065	10.832
	W365	9.837	9.272	94.253
	STAY48	9.837	1.444	14.683
	STAY54	9.837	0.774	7.866
	STAY72	9.837	0.125	1.274

	TRAITS	ΔG (STAY48)	RC	ER (%)
STAY48	LFT	0.118	0.041	34.923
	REFT	0.118	0.063	53.149
	LEA	0.118	0.005	3.914
	W365	0.118	0.070	59.422
	STAY54	0.118	0.103	87.526
	STAY72	0.118	0.090	76.624

	TRAITS	ΔG (STAY54)	RC	ER (%)
STAY54	LFT	0.128	0.025	19.236
	REFT	0.128	0.040	31.430
	LEA	0.128	0.000	0.104
	W365	0.128	0.050	38.806
	STAY72	0.128	0.129	100.796

	TRAITS	ΔG (STAY72)	RC	ER (%)
STAY72	LFT	0.413	0.018	4.427
	REFT	0.413	0.068	16.497
	LEA	0.413	0.012	2.889
	W365	0.413	0.006	1.577

Abbreviations: LEA, loin eye area; LFT, subcutaneous fat thickness in the loin; REFT, subcutaneous fat thickness in the rump; STAY48, stayability at 48 months; STAY54, stayability at 54 months; STAY72, stayability at 72 months; W210, standardised weight at 210 days; W365, standardised weight at 365 days; W450, standardised weight at 450 days.

The genetic gain for W450 was 9.837 kg through direct selection. However, through correlated response via indirect selection for LFT, REFT, LEA, STAY48, STAY54, STAY72 and W365, we obtained estimates ranging from 0.125 to 9.272 kg (resulting in a relative selection efficiency ranging from 1.274 to 94.253%). In other words, indirectly selecting for W450 through LFT, REFT, LEA, STAY48, STAY54 and STAY72 will result in a lower gain for W450. This demonstrates that indirect selection for W450 through LFT, REFT, LEA, STAY48, STAY54 and STAY72 was not efficient when compared to direct selection for W450, which may be justified by the low genetic correlation estimates between these traits (−0.330 to 0.182).

The genetic gain for STAY48 was 0.118 months through direct selection. However, through correlated response via indirect selection for LFT, REFT and LEA, we obtained estimates ranging from 0.005 to 0.103 months, resulting in a relative selection efficiency ranging from 3.914 to 59.422%. In other words, indirect selection for STAY48 through LFT, REFT, LEA and W365 will result in a lower gain for STAY48. This demonstrates that indirect selection for STAY48 through LFT, REFT, LEA and W365 was not efficient when compared to direct selection for STAY48, which may be justified by the low genetic correlation estimates between these traits (−0.324 to 0.272).

The genetic gain for STAY54 was 0.128 months through direct selection. However, through correlated response via indirect selection for LFT, REFT, LEA and W365, we obtained estimates ranging from 0.000 to 0.050 months, resulting in a relative efficiency of 0.104 to 38.8065%. Thus, indirectly selecting for the STAY54 trait through LFT, REFT, LEA and W365 will result in a lower genetic gain. In this context, indirect selection through LFT, REFT, LEA and W365 was not more efficient when compared to direct selection for STAY54, primarily due to the low genetic correlation estimates between the traits, ranging a posteriori from −0.221 to 0.168. Stefani et al. (2018) reported a negative correlation of lower magnitude (−0.25) between STAY60 and milk production in Holstein cows. This would suggest that higher milk yields associate with reduced STAY60, and maximum relative efficiency would be achieved by directly selecting for STAY60. Indirect selection for STAY54 through STAY72 showed a genetic gain of 0.129 months and a relative efficiency of 100.796%, illustrating that indirect selection for STAY54 through STAY72 is more efficient.

The genetic gain for STAY72 was 0.413 months (through direct selection). However, through correlated response via indirect selection for LFT, REFT, LEA and W365, we obtained estimates ranging from 0.006 to 0.068 months, resulting in a relative efficiency of selection ranging from 1.577 to 16.497%. Thus, indirect selection for STAY72 by means of LFT, REFT, LEA and W365 will result in lower genetic gains for the trait. Thus, indirect selection for LFT, REFT, LEA and W365 would not be more efficient than direct selection for STAY72, primarily due to the low genetic correlation estimates between these traits, which ranged from −0.034 to 0.108. Carvalho et al. (2023) considered STAY as the trait for female longevity (LG) of Murrah buffaloes and measured the correlation with milk production accumulated at 305 days (P305). They reported a correlation of 0.77 between both traits and a 7% increase in LG genetic gain under indirect selection for W305. Thus, higher estimates of the genetic correlation may result in more efficient indirect selection for low heritability traits of economic importance.

## 4 Conclusion

Direct genetic selection for W210, W365, W450, LFT and REFT was the result of sizeable genetic variability in single‐trait analyses. Heritability estimates for the different STAYs and LEA suggested that the major part of the variation in these traits was due to environmental and non‐additive genetic factors. However, the economic importance of these traits justifies their selection. Genetic trends for W365, W450, REFT and STAY72 were positive and significant, indicating substantial improvements over the years. Indirect selection for W450, through traits such as LEA, LFT, REFT, STAY48, STAY54 and STAY72, would not be effective due to low genetic correlations. Similarly, indirect selection for STAYs through LEA and LFT would also be inefficient, as genetic gain would be lower than direct selection. However, selection for W365 and REFT may positively influence STAYs, as carcass finishing affects body condition, an important biological component. The correlated responses for W450 and STAYs were lower than direct genetic gains, suggesting greater effectiveness of direct selection. The results of this study reinforce the importance of understanding heritabilities, genetic trends, correlations and genetic gains among economically important traits, aiming to optimise genetic selection programmes in Nelore, considering the interaction between genetic and environmental factors to drive animal genetic improvement.

## Conflicts of Interest

The authors declare no conflicts of interest.

## Data Availability

The data supporting the findings of this study are available from the Associação Nacional de Criadores e Pesquisadores (ANCP). However, restrictions apply to their availability, as they were used under licence for this study. The authors cannot provide access to the data, as they are owned by the ANCP, and no permission has been granted for sharing.
